# An In Vitro Model of the Blood–Brain Barrier for the Investigation and Isolation of the Key Drivers of Barriergenesis

**DOI:** 10.1002/adhm.202303777

**Published:** 2024-08-05

**Authors:** Christina Schofield, Stylianos Sarrigiannidis, Alejandro Moran‐Horowich, Emma Jackson, Aleixandre Rodrigo‐Navarro, Tom van Agtmael, Marco Cantini, Matthew J. Dalby, Manuel Salmeron‐Sanchez

**Affiliations:** ^1^ Centre for the Cellular Microenvironment University of Glasgow Glasgow G11 6EW UK; ^2^ School of Cardiovascular and Metabolic Health University of Glasgow Glasgow G12 8TA UK; ^3^ Institute for Bioengineering of Catalonia (IBEC) The Barcelona Institute for Science and Technology (BIST) Barcelona 08028 Spain; ^4^ Institució Catalana de Recerca i Estudis Avançats (ICREA) Barcelona Spain

**Keywords:** BBB, ECM, electrospinning, growth factors, in vitro model

## Abstract

The blood–brain barrier (BBB) tightly regulates substance transport between the bloodstream and the brain. Models for the study of the physiological processes affecting the BBB, as well as predicting the permeability of therapeutic substances for neurological and neurovascular pathologies, are highly desirable. Existing models, such as Transwell utilizing‐models, do not mimic the extracellular environment of the BBB with their stiff, semipermeable, non‐biodegradable membranes. To help overcome this, we engineered electrospun membranes from poly L‐lactic acid in combination with a nanometric coating of poly(ethyl acrylate) (PEA) that drives fibrillogenesis of fibronectin, facilitating the synergistic presentation of both growth factors and integrin binding sites. Compared to commercial semi‐porous membranes, these membranes significantly improve the expression of BBB‐related proteins in brain endothelial cells. PEA‐coated membranes in combination with different growth factors and extracellular protein coatings reveal nerve growth factor (NGF) and fibroblast growth factor (FGF‐2) caused formation of better barriers in vitro. This BBB model offers a robust platform for studying key biochemical factors influencing barrier formation that marries the simplicity of the Transwell model with the highly tunable electrospun PEA‐fibronectin membranes. This enables the generation of high‐throughput drug permeability models without the need of complicated co‐culture conditions.

## Graphical abstract

The blood–brain barrier (BBB) tightly regulates substance transport between the bloodstream and the brain. Here a simple model of the BBB that allows culture of endothelial cells on growth‐factor functionalised membranes is introduced. This novel in vitro model of the BBB offers a robust platform for studying key barriergenic biochemical factors influencing barrier formation without the use of the complicated co‐culture conditions.

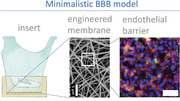

## 1 Introduction

The blood–brain barrier (BBB) acts as a near impermeable barrier that selectively controls the exchange of molecules between the brain and the bloodstream and protects the central nervous system (CNS) from pathogens and neurotoxic substances.^[^ ^1^ ^]^ It is comprised of brain endothelial cells (BMECs), pericytes, astrocytic end feet, and their shared basement membranes (BMs).^[^ ^2^ ^]^ The BM within the BBB is largely composed of collagen IV, laminin, and heparan sulfate proteoglycans, among other proteins, which allow for the adhesion of cells and help direct their differentiation through the regulation of their crosstalk with the surrounding microenvironment.^[^ ^3^ ^]^

As the global average age increases, significant efforts have been made to offset the enormous social and economic burden of both neurological and neurovascular diseases, which increase in prevalence within older populations.^[^ ^4^, ^5^ ^]^ A substantial portion of this endeavor is the modeling of the BBB to both better understand the microenvironment and model drug permeability and disease.^[^ ^6^ ^]^ The BBB poses a formidable challenge to the efficient delivery of therapeutics aimed at targeting the brain, further compounded by the lack of a priori knowledge of the kinetics of drug delivery to the brain, as well as the interspecies differences in expression of human‐relevant phenotypes.^[^ ^7^ ^]^ To help overcome this, there is a need for an effective in vitro BBB model for disease and drug transport studies that is robust and simple to use. Further, the exact molecular mechanisms and cellular signaling pathways vital to the establishment and maintenance of the barrier remain largely unknown.^[^ ^6^ ^]^

Regardless of the cells that are being used in models, the potential of BMECs is curtailed in vitro by the artificial constructs upon which they are grown.^[^ ^8^, ^9^, ^10^, ^11^, ^12^ ^]^ This applies to immortalized BMECs, primary cells, and the now‐favored endothelial cells‐like induced pluripotent stem cells (iPSCs). Chief among these constructs are the Transwell, MilliCell, or similar cell culture inserts that reduce barriers to their most simplistic form. These commercial inserts consist of a single‐use semiporous membrane made from materials such as polycarbonate (PC) and polytetrafluoroethylene (PET), and can cost ≈$300 per plate.^[^ ^13^, ^14^ ^]^ These flat, featureless, stiff semi‐porous membranes lack the ability to allow cells to dynamically interact with and restructure their local environments, leading to the formation of artificial cellular function.^[^ ^8^, ^15^ ^]^ Efforts to simulate a more realistic representation of the BBB by reproducing the circulatory environment have led to the hollow fiber model and other microfluidic BBB systems.^[^ ^16^, ^17^, ^18^, ^19^, ^20^ ^]^ However, their complex co‐cultures limits their throughput capacity and adoption by the research communities, and makes them less practical for pharmaceutical use. This underscores a need for a novel robust, yet simple, BBB model to understand function, disease, and screening CNS drugs. This paper contributes by introducing a simple functionalized membrane that allows monoculture of functional endothelial cells to recapitulate barrier properties.

Poly L‐lactic acid (PLLA) is an established biodegradable polymer in biomedical engineering used in in vitro and in vivo applications for its biocompatibility, processibility, and controlled degradation.^[^ ^21^, ^22^ ^]^ However, PLLA lacks bioactivity with limited cell adhesion. Thus, it is often modified using surface modifications,^[^ ^23^, ^24^ ^]^ improving the cell‐material interfaces and enhancing the cell functional properties of the coated material.^[^ ^25^ ^]^ Plasma polymerization enables inexpensive coating of complex 3D structures with highly cross‐linked, nanometric thin polymer coatings, that do not alter the mechanical properties of the substrate material, with controlled thickness of the coating to the tens of nanometers.^[^ ^24^, ^25^, ^26^, ^27^ ^]^ Our group has previously shown that poly(ethyl acrylate) (PEA) induces the spontaneous unfurling of globular fibronectin (Fn), a major component of the extracellular matrix (ECM), into an nanonetwork, revealing its integrin and growth factor (GF) binding domains.^[^ ^23^, ^24^, ^28^, ^29^, ^30^, ^31^ ^]^ As these binding sites are in close proximity, this drives synergistic signaling to enhance downstream mitogenic capabilities of both signals. When Fn is adsorbed on PLLA, it remains globular and does not expose the integrin and GF binding sites.^[^ ^23^, ^32^, ^33^ ^]^ Plasme polymerized PEA (pPEA) also promotes Fn fibrillogenesis, activating signaling with ultra‐low doses of GFs such as bone morphogenic protein 2 that increases osteogenesis in bone formation models.^[^ ^31^ ^]^

Therefore, to make a BBB model that combines the simplicity of the Transwell model with the barrier‐inducing biochemical and biomechanical characteristics of the native ECM, we developed a nanofibrous membrane that combines the mechanical properties of PLLA with the GF and integrin binding site presentation resulting from PEA. Following an in‐depth structural characterization of the electrospun fibers, the mechanical and biological capabilities of the membrane were determined using brain endothelial cell monocultures. We note that our model does not include other relevant cells that are typically used in BBB modeling such as astrocytes and pericytes. Yet we want to investigate the ability of highly engineered membranes to promote the formation of the brain endothelial monolayer. We show that pPEA‐Fn coatings increase expression of BBB‐relevant proteins specifically on electrospun membranes rather than on MilliCell cell culture inserts. Thus by showing our model outcompetes the traditional transwell inserts while maintaining its simplicity, these results demonstrate the potential for this in vitro model to isolate and characterize potential barriergenic signals and for it to be further developed for drug permeability testing and disease modeling for the BBB research community.

## 2 Results and Discussion

### 2.1 Electrospun Membrane Fabrication

The first step in the production of the in vitro model was the development of a 2.5D cell culture scaffold that more closely mimicked the native BBB basement membrane (BM) than commercial cell culture inserts, such as the Transwell and the MilliCell, without the loss of ease of use. To achieve this, we utilize PLLA, which is FDA‐approved for pharmaceutical and biomedical applications, which was electrospun to generate a three‐dimensional fibrous structure, resembling that of the BM.^[^ ^34^, ^35^, ^36^ ^]^ While PLLA has the positive attributes of being both biocompatible, readily available, economic, and highly processible, it lacks bioactivity, limiting cell adhesion.^[^ ^23^ ^]^ In line with work previously published by our group, these PLLA electrospun membranes were coated with a nanometric coating of plasma PEA (pPEA), which has the remarkable chemical and mechanical properties which causes the extracellular protein, fibronectin (Fn), to undergo fibrillogenesis.^[^ ^23^, ^24^, ^29^, ^30^, ^31^ ^]^ This involves the unfolding of the Fn molecule upon adsorption onto PEA surfaces, presenting the heparin binding and cell adhesion sites, whereas on the PLLA, Fn is adsorbed in the globular form. The revealing of these biological binding sites is favorable for the enhancement of the biological activity of the substrate through synergistic integrin‐growth factor signaling.^[^ ^23^, ^32^, ^33^, ^37^ ^]^

**Figure 1:**
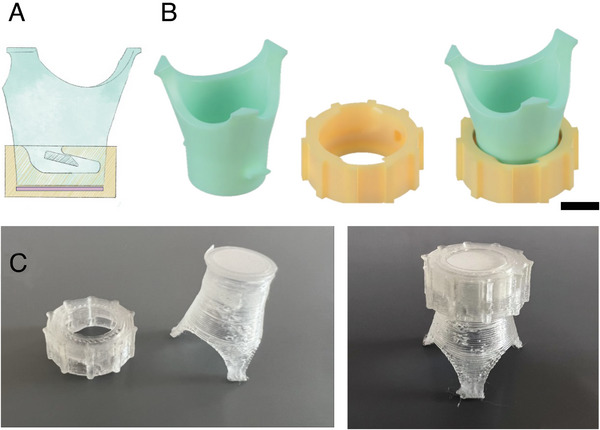
Custom designed membrane holder for cell culture in 24 well plates. A) Illustration showcasing the fastening mechanism between insert base (blue) and cap (yellow), and highlighting the position of membrane (pink). Membranes are sandwiched between the cell culture insert base. B) Renders of inserts. From left to right: cell culture insert base, insert cap, insert shown assembled. C) images of 3D printed inserts, with cap and membrane‐mounted insert separated (left) and mounted (right). Scale bar represents 5 mm.

**Figure 2:**
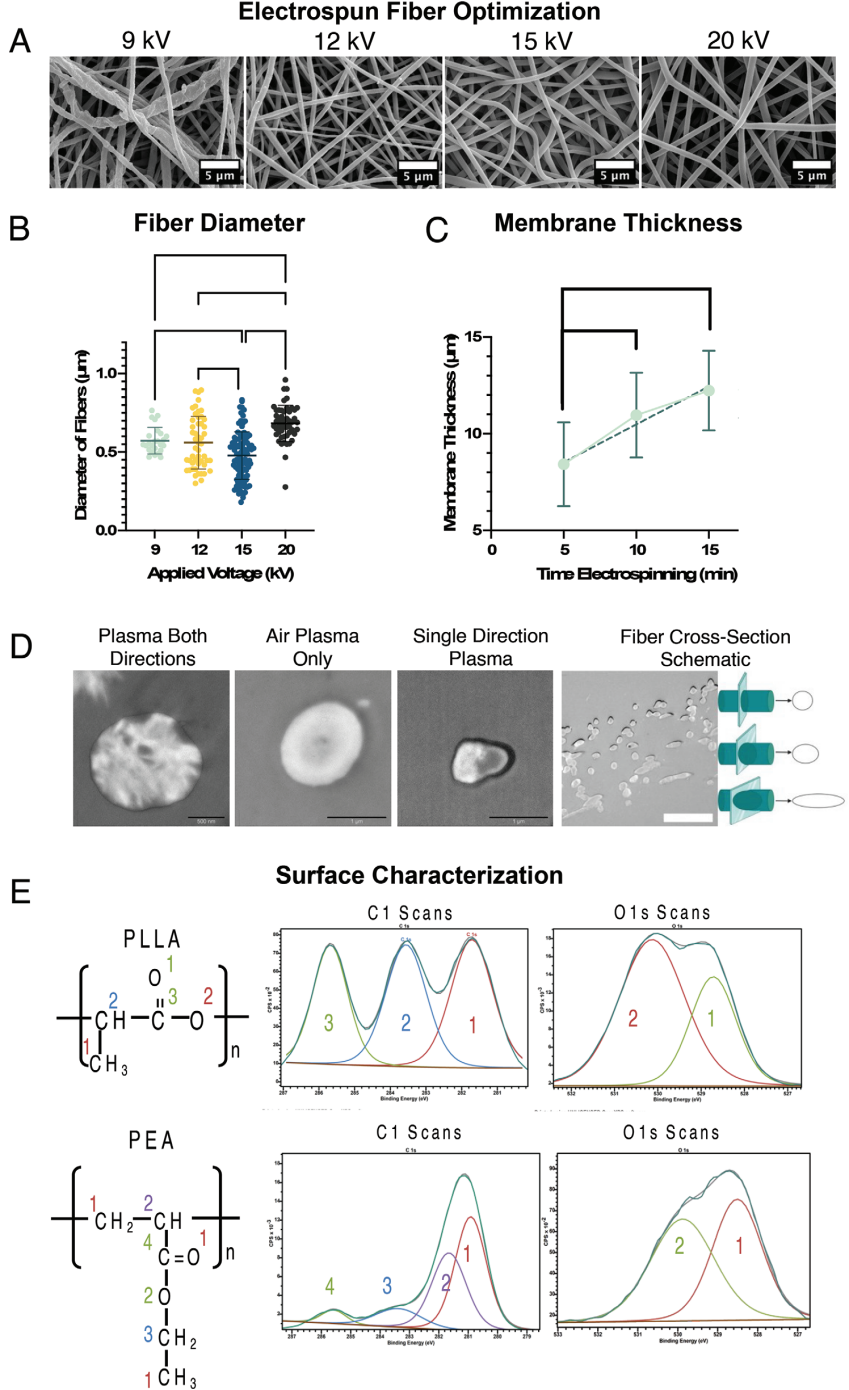
Electrospun membrane characterization: A) SEM images comparing electrospun PLLA nanofibers produced by changing the applied voltage during the electrospinning process, and B) the resulting fiber diameters measured from the SEM images. C) The change in membrane thickness of the electrospun mats in relation to the length of time of the electrospinning as measured from TEM images. D) TEM images of fiber cross‐sections. From left to right, TEM image of fiber which has only been treated with air plasma, sample section from membrane treated with ethyl acrylate plasma from both membrane faces, fiber which is treated with air plasma and monomer plasma under the protocol used for the model, and a schematic showing the cross‐section of an entire membrane and explanation for how fibers are cross‐sectioned, and the resulting oblong shapes which might arise. For images showing a single fiber diameter, the scale bar represents 500 nm, for the cross‐section of the membrane used in the schematic, the scale bar represents 5 µm. E) XPS surface characterization of PLLA (top) and PLLA‐pPEA (bottom) membranes. Schematic for PLLA and PEA are given, with carbons and oxygens numbered in accordance with the peaks shown in the C1s and O1s scans. ^*^ *p* < 0.05, ^**^ *p* < 0.01, ^***^ *p* < 0.001, ^****^ *p* < 0.0001.

To best handle the electrospun membranes for cell culture, custom cell culture inserts were designed and 3D printed (**Figure** **1**) The electrospinning produced randomly oriented, defect‐free fibers, homogenous in nature and with a smooth surface (**Figure** **2**A). In order for the electrospun membrane to best represent the native BM and ECM, fiber diameter was optimized to closely resemble that of collagen‐IV, a main constituent of the BBB BM, as cell differentiation and adhesion can be affected by fiber diameter.^[^ ^38^, ^39^ ^]^ As the fibril diameter of collagen‐IV ranges from 50 to 500 nm, the 15 kV applied voltage and 20 cm gap distance were used after confirming that the average fiber diameter was 479.5 nm ± 150.5 nm (Figure 2B). For the cultivation of a tight endothelial barrier, the current literature additionally states smaller pore sizes improve the barriergenic nature of scaffolds.^[^ ^40^, ^41^, ^42^ ^]^ The correlation between smaller diameter fibers and decrease in pore size was confirmed, for which the above‐mentioned electrospinning conditions produced pores in the range of 0.43 µm^2^ ± 0.68 µm^2^. As such, the use of the 20 cm gap distance and 15 kV applied voltage provides both improved physiological fibril‐like nature at the individual fiber level, but additionally has improved porosity, which should additionally increase the barrier‐forming capabilities of the resulting membrane by decreasing cell infiltration of the membrane and encouraging monolayer formation.^[^ ^43^ ^]^

In vivo, the BM provides support to the BMECs, but also acts as a separation between BMECs and the surrounding cells of the NVU. Consequently, to best replicate the native BM of the BBB, the electrospun membranes were fabricated to be as thin as possible. To accommodate this, membranes were fabricated to be 12.2 µm ± 2.1 µm, which is comparable to the thickness of the Transwell inserts (Figure 2C).^[^ ^44^ ^]^ We note that the thickness of our membranes is significantly higher than the BM. The handling of these membranes was enhanced through the use of custom‐designed and 3D‐printed cell culture inserts (Figure 1).

### 2.2 Poly (Ethyl Acrylate) Coating Characterization

X‐ray photoelectron spectroscopy (XPS) measurements were used to compare the chemical composition of the surfaces of uncoated PLLA electrospun membranes and pPEA‐coated membranes, which were analyzed by fitting the carbon (C1s) and oxygen (O1s) spectra (Figure 2E). These revealed characteristic peaks for pPEA on the coated samples, an indicator of successful coating, with a diminished peak for both the carboxyl (COO) and ester bond (C) found on the PEA side chain, compared to the expected peak sizes for PEA. Plasma polymerization is known to cause fragmentation in the PEA side chains, leading to the loss of peaks for the carboxyl and ester bonds and unknown rearrangements.^[^ ^23^, ^24^ ^]^ Regardless of side chain loss, pPEA is still shown to drive Fn fibrillogenesis within the literature.^[^ ^31^ ^]^ O1s spectra were fitted for the carboxyl (COO) and ester bond (CO) found in both PLLA and PEA and were used to confirm the identification of both of them. TEM was used to measure membrane thickness and to explore whether pPEA could be visualized on the fibers. The revealed dark rings surrounding pPEA‐coated electrospun fibers, were not seen in fibers that only received air plasma (Figure 2D). Then, it was hypothesized that side chain loss of pPEA would result in a denser packing of remaining side chains, which could result in these darker areas in TEM. These rings were weighted towards one side in membranes coated from a single direction, and more evenly weighted in membranes coated from both sides of the membrane.

### 2.3 Physical and Mechanical Characterization of Electrospun Membranes

**Figure 3:**
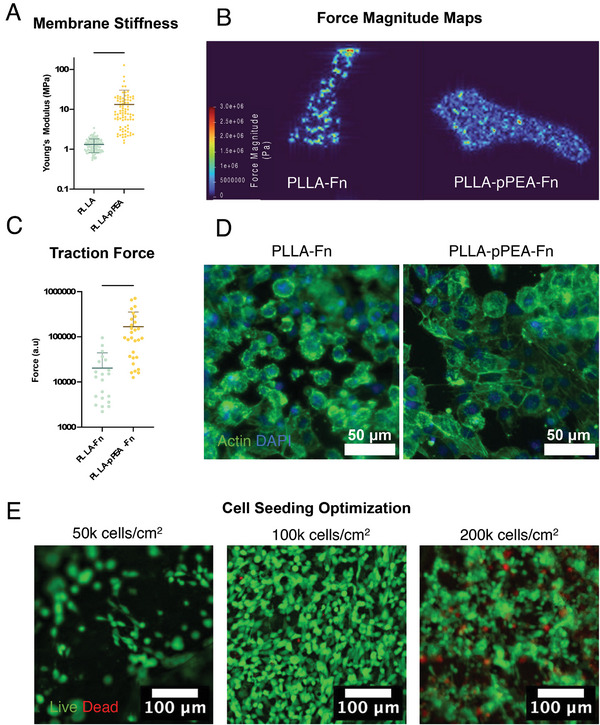
Effect of pPEA coating on PLLA membrane mechanical properties and the resulting interaction with cells. A) Membrane stiffness measured by AFM nanoindentation of PLLA and PLLA‐pPEA membranes. B) Force magnitude map generated by MSC cells seeded on PLLA and PLLA‐pPEA membranes after 24 h and C) force magnitude calculated over 20 cells. D) Immunofluorescence of hCMEC/D3 BMEC cells seeded on PLLA‐Fn and PLLA‐pPEA‐Fn membranes, imaged after 24 h. E) LIVE/DEAD™ stain of hCMEC/D3 cells seeded on PLLA‐pPEA‐Fn membranes after 24 h for monolayer optimization. ^****^ *p* < 0.0001.

To evaluate the effect of pPEA‐coating on the mechanical properties of the PLLA electrospun membranes, including the ability of cells to perform forces on the membranes, atomic force microscope (AFM) nanoindentation and traction force microscopy (TFM) was used (**Figure** **3**). Nanoindentation revealed an increase in the Young's modulus of the fibers following pPEA coating (Figure 3A). Particularly in the pPEA‐coated samples, distinct populations of stiffness were seen, likely arising from where the cantilever samples the membranes – in more porous regions, in regions where many fibers lay on top of one another, or a single fiber. The increased stiffness of the pPEA‐coated fibers is thought to arise from the adhesion of individual fibers together by the coating, preventing the independent sliding of fibers over one another in response to forces. After confirming the increase in membrane stiffness, TFM was used to evaluate whether this affected the ability of cells to manipulate fibers (Figure 3B,C). The force map produced by cells seeded on PLLA membranes coated in Fn showed displacement of the beads occurs in “lines”. As the beads are constrained within the fibers, this could be a visual representation of the movement of individual fibers by cells. The PLLA‐Fn force magnitude map is significantly different than those produced by cells seeded on PLLA‐pPEA‐Fn membranes, in which the areas of force have a nosier visualization rather than distinct areas of displacement. We have expressed forces in a.u. (rather than nN) to emphasize that the force magnitude measured using electrospun membranes has caveats, as the theory supporting TFM supposed the free movement of beads within a three‐dimensional environment, by directly comparing the results of PLLA‐Fn and PLLA‐pPEA‐Fn with one another. Our results support the hypothesis that the pPEA coating acts as an adherent between fibers, making them more difficult to displace and the overall membrane stiffer.^[^ ^45^ ^]^ Alternatively, the force magnitude may be larger on the pPEA‐Fn coated membranes, as Fn is in its extended form, allowing for better adhesion from the cells and therefore a greater ability to exert force on their local environment. This is seen in Figure 2D, in which the f‐actin immunofluorescence for hCMEC/D3 cells is shown on PLLA‐Fn and PLLA‐pPEA‐Fn membranes. When seeded on the pPEA‐coated membranes, the cells are larger with longer actin filaments in comparison to the rounder PLLA‐Fn seeded cells. The potential of having an electrospun membrane platform with good bifunctionality and a tunable stiffness through the deposition of a controlled thickness of pPEA is an exciting future avenue for tissue engineering, and whether a lower power or time of pPEA coating would have less of an effect on the electrospun membranes is an avenue for future research. This is further supported by the work of Alba‐Perez et al., in which they show that lower power coatings of pPEA than those used in this paper are still able to induce fibrillogenesis of Fn.^[^ ^24^ ^]^ We note that our membranes are significantly stiffer than the brain and that it is not our intention to mimic the mechanical properties of the physiological BBB but to provide an environment that supports the functionality of endothelial brain cells.

hCMEC/D3 seeding was established through LIVE/DEAD staining cells 24 h after seeding, which established 1 × 10^5^ cells cm^−2^ as the best condition (Figure 3E). As the final evolution of this model will be a highly tuned high‐throughput drug permeability model, a quick establishment of a monolayer is necessary. By removing the internal insert volume 12–24 h after seeding, it was also shown that the development of non‐monolayers could be prevented, despite the high seeding density.

**Figure 4:**
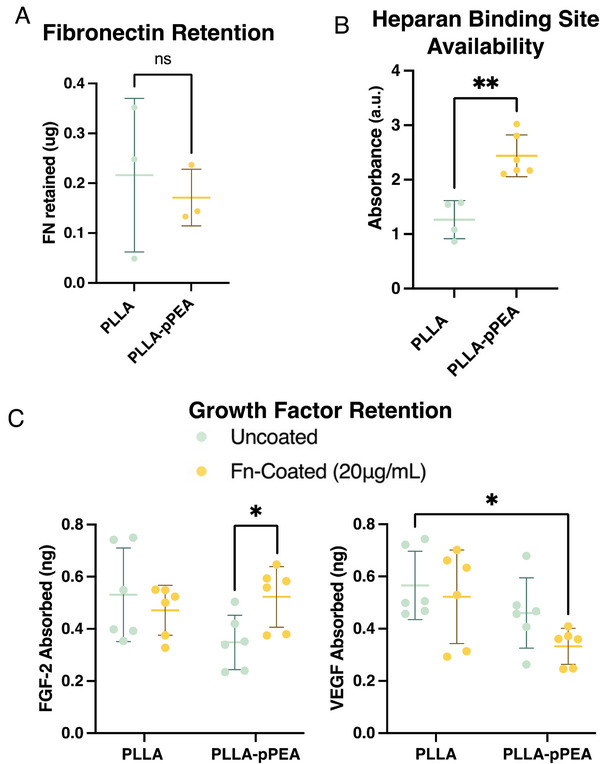
Characterization of the pPEA, fibronectin, and growth factor interactions. A) Fibronectin retained on membranes, as measured by microBCA assay. B) Result from indirect ELISA on heparan binding site availability on fibronectin adsorbed on PLLA and PLLA‐pPEA membranes. C) Growth factor retention on PLLA, PLLA‐Fn, PLLA‐pPEA, and PLLA‐pPEA‐Fn membranes, with results from sandwich ELISAs for FGF‐2 (left) and VEGF (right) ^*^ *p* < 0.05, ^**^ *p* < 0.01.

To demonstrate the formation of non‐globular Fn adsorbed onto pPEA‐coated membranes, we compared the availability of the heparin binding site and the ability of coated and uncoated membranes to retain GFs using ELISAs (**Figure** **4**). These results show that there is an equal amount of Fn adsorption on both the uncoated and pPEA‐coated PLLA membranes (Figure 4A). Furthermore, there is an increased availability of the heparin binding site and enhanced retention of FGF‐2 on PLLA‐pPEA membranes (Figure 4B,C). Although one might argue that the increased retention of Fn onto pPEA‐coated surfaces would also result in increased retention of these GFs, provided the results in Figure 4A, showing equal Fn retention, and Figure 4B, which illustrates increased heparin binding site availability, the pPEA‐Fn coating has been shown to enhance the bioactivity of PLLA electrospun membranes through pPEA‐induced fibrillogenesis of Fn, rather than an increased retention of Fn on PLLA‐pPEA membranes. There was a correlation between the availability of the heparin binding site on Fn on pPEA and the retention of growth factors (Figure 4C).^[^ ^37^ ^]^

### 2.4 Cultivation of BMECs on Electrospun Membrane

**Figure 5:**
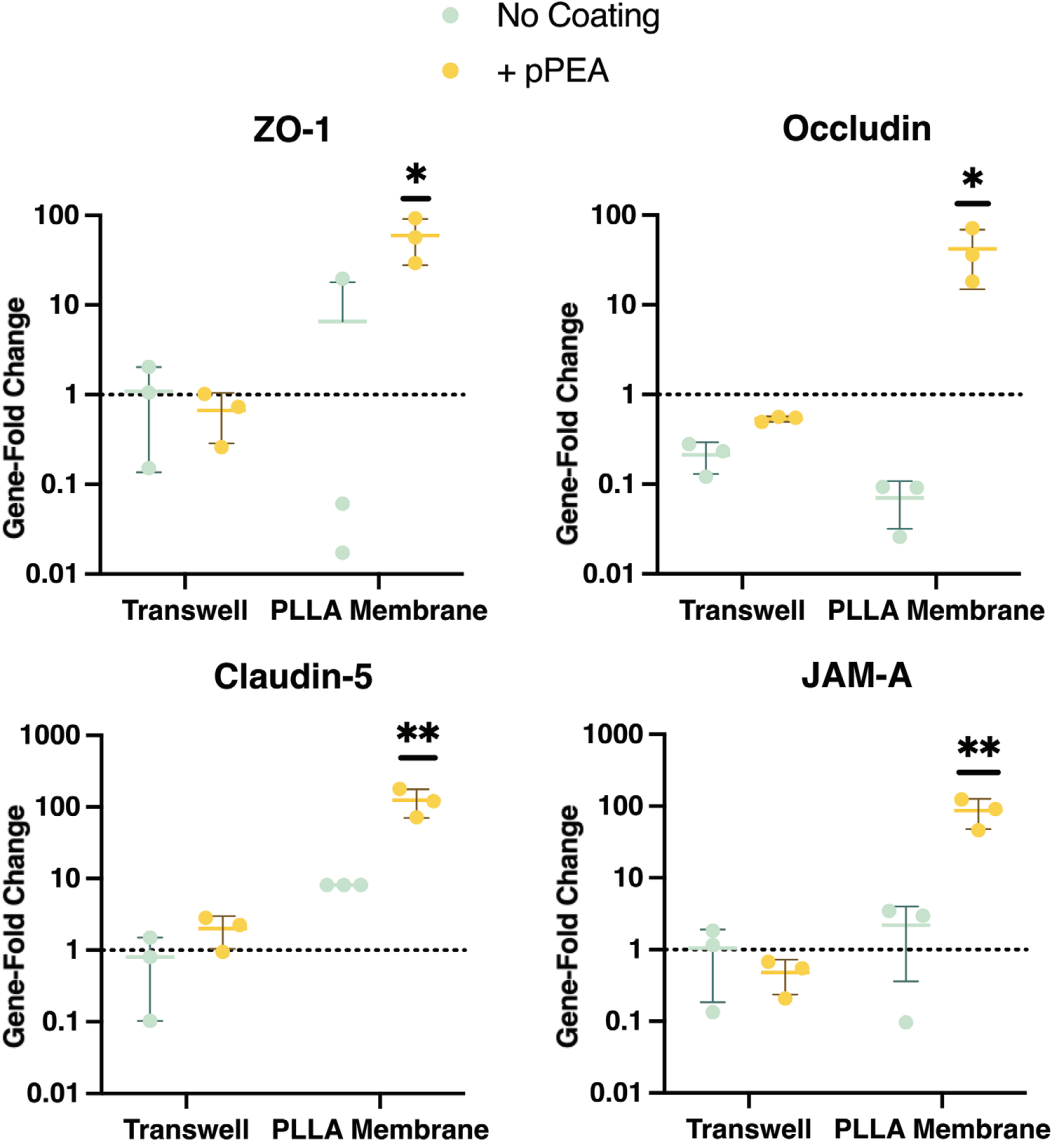
Comparison of pPEA‐Fn coating on Transwell cell culture inserts and PLLA electrospun membranes. hCMEC/D3 cells were seeded for 10 days on fibronectin‐coated membranes before gene expression was acquired for ZO‐1, occludin, claudin‐5, and JAM‐A. ^*^ *p* < 0.05, ^**^ *p* < 0.01.

The ability of the electrospun membranes, and the pPEA coating, was compared to MilliCell cell culture inserts for their ability to change the expression of tight junction (TJ), and TJ‐associated proteins was tested on immortalized BMEC cell line, hCMEC/D3 cells, utilizing FGF‐2 at a 100 ng mL^−1^ coating as a stand‐in growth factor. Of considerable interest, PLLA‐pPEA‐Fn‐FGF‐2 significantly increased the expression of all tested genes in comparison to the other insert‐coating conditions, and the addition of pPEA coating showed a greater change in expression when electrospun membranes were coated compared to the MilliCell cell culture inserts (**Figure** **5**). A previous study has similarly found improved TJ formation on their gelatin‐based electrospun membranes in comparison to commercial Transwell membranes.^[^ ^12^ ^]^ Electrospun membranes have previously been used to model the BBB using a mixture of immortalized and induced pluripotent stem cell lines, although many of these fail to attain the high transendothelial electrical resistance (TEER) associated with a complete barrier, with few reaching 100 Ω cm^2^.^[^ ^12^, ^46^, ^47^, ^48^, ^49^ ^]^ Our model was able to produce high TEER values, with monolayers grown on pPEA‐Fn‐FGF‐2 coated membranes with an average TEER value of 738 Ω cm^2 ^± 480, although the large variability in TEER values could be improved with more homogenized membrane thicknesses and controls for membrane tearing.^[^ ^50^ ^]^

**Figure 6:**
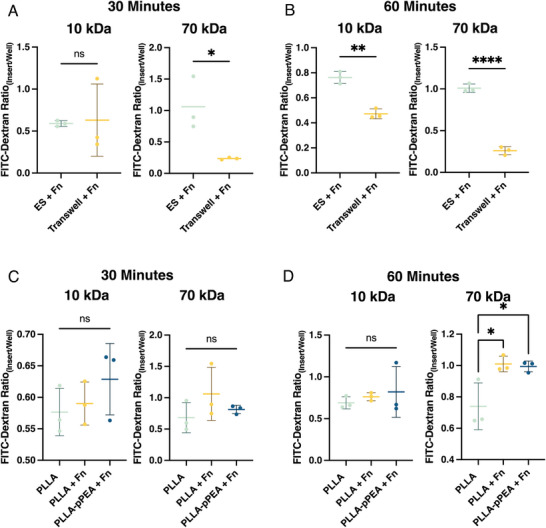
Impact of Membrane Conditions on Barrier Permeability. The permeability is quantified as the ratio of FITC‐dextran signal between the insert and the well at 30‐ and 60‐min post‐addition of either 10 kDa or 70 kDa FITC‐dextran to the insert. A) and B) compare the permeability of electrospun membranes (ES) and Transwell inserts with fibronectin at 30 and 60 min, respectively. C) and D) compare uncoated electrospun membranes (PLLA) with and without fibronectin, and pPEA‐PLLA membranes with fibronectin, at 30 and 60 min, respectively. ^*^ *p* < 0.05, ^**^ *p* < 0.01, ^****^ *p* < 0.0001.

The effect of membrane conditions on barrier permeability was also examined with low FGF‐2 media (**Figure** **6**). PLLA‐Fn inserts had a significantly lower permeability (displayed here as a greater ratio of FITC‐dextran inside the insert compared to the FITC‐dextran that passes the cell barrier into the well) compared to Transwell inserts coated with Fn under the same conditions for 70 kDa FITC‐dextran at all time points and 10 kDa at the 60‐min mark (Figure 6A,B). This trend is also seen in the 10‐day permeability results when the inserts and Transwell membranes are incubated with NGF and FGF‐2 prior to cell seeding, in which the electrospun membranes coated with pPEA and Fn had significantly lower permeability than the Fn‐coated Transwell membranes (30‐min mark: 10 kDa *p < 0.05*, 70 kDa *p < 0.0005*; 60‐min mark: 10 kDa *p < 0.05*, 70 kDa *p < 0.005*).

Within the PLLA electrospun membrane conditions, the addition of Fn and pPEA‐Fn did not result in a statistically significant reduction in permeability, except for the 70 kDa FITC‐dextran measurements depicted in Figure 6D. In this specific case, both PLLA‐Fn and PLLA‐pPEA‐Fn showed significantly lower permeability than PLLA alone (p < 0.05). This indicates that while PLLA‐Fn provides an effective barrier, the further inclusion of pPEA‐Fn does not substantially enhance the permeability resistance for most conditions tested, which is to be expected, as the purpose of presenting the Fn to cells in a pre‐stretched nanonetwork, as occurs when adsorbed onto PLLA‐pPEA surfaces, is for the binding and effective presentation of GFs, and cells are capable of mediating the formation of Fn fibrils post‐seeding.^[^ ^24^ ^]^

**Figure 7:**
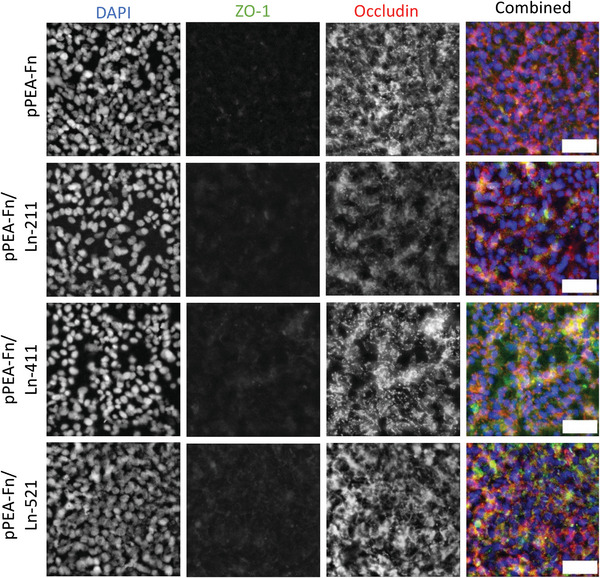
Comparison of different laminin isoforms co‐adsorbed with fibronectin of ZO‐1 and occludin immunofluorescence of hCMEC/D3 cells cultured on PLLA‐pPEA‐FGF‐2 membranes. hCMEC/D3 cells were cultured for 10 days on membranes.

**Figure 8:**
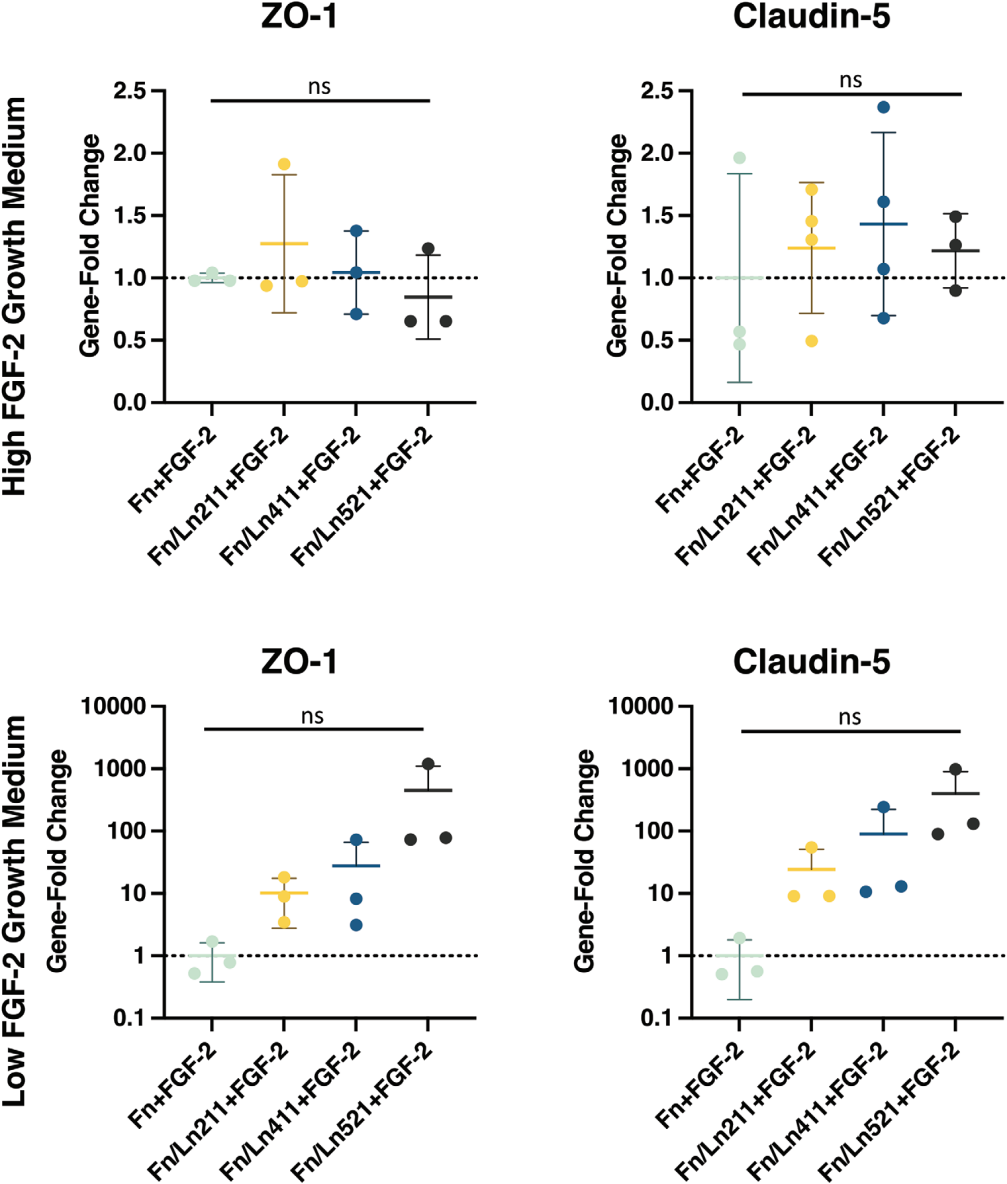
Comparison of gene‐expression of ZO‐1 and claudin‐5 of hCMEC/D3 cells cultured on PLLA‐pPEA membranes coated with fibronectin and laminin with FGF‐2. Gene expressions displayed on the top row were acquired from cells cultured in high FGF‐2 concentration commercial cell medium, and low FGF‐2 medium on the bottom row. hCMEC/D3 cells were cultured for 10 days.

There are few individual studies investigating the effect of different BM protein coatings on barrier formation in vitro. However, according to the work of Tilling et al., co‐adsorption of laminin with Fn on membranes significantly increases the barriergenic potential of Transwell‐based models.^[^ ^51^ ^]^ Laminin‐211, −411, and −521 were each co‐adsorbed with Fn and both gene expression and IF of occludin and ZO‐1 were compared (**Figure** **7**). In our study, laminin did not have a statistically significant effect on occludin and ZO‐1 expression, although laminin‐521 did produce a speckled appearance of the occludin, where it clustered along the cell–cell junction in broken repeats. Claudin‐5 and ZO‐1 gene expression for these conditions were additionally evaluated, and tested in both commercially supplemented cell media (high FGF‐2) and low FGF‐2 media. (**Figure** **8**). Likewise, no significant difference between PLLA‐pPEA‐Fn‐FGF‐2 or conditions in which laminin had been co‐adsorbed could be seen in the gene expression, either in commercial cell medium or low FGF‐2 concentration cell medium. Laminin within the Tilling et al. study may lead to enhanced Fn spreading for the presentation of its cell adhesion sites, which is overcome in this model through the use of pPEA, making the addition of laminin redundant.^[^ ^51^ ^]^

**Figure 9:**
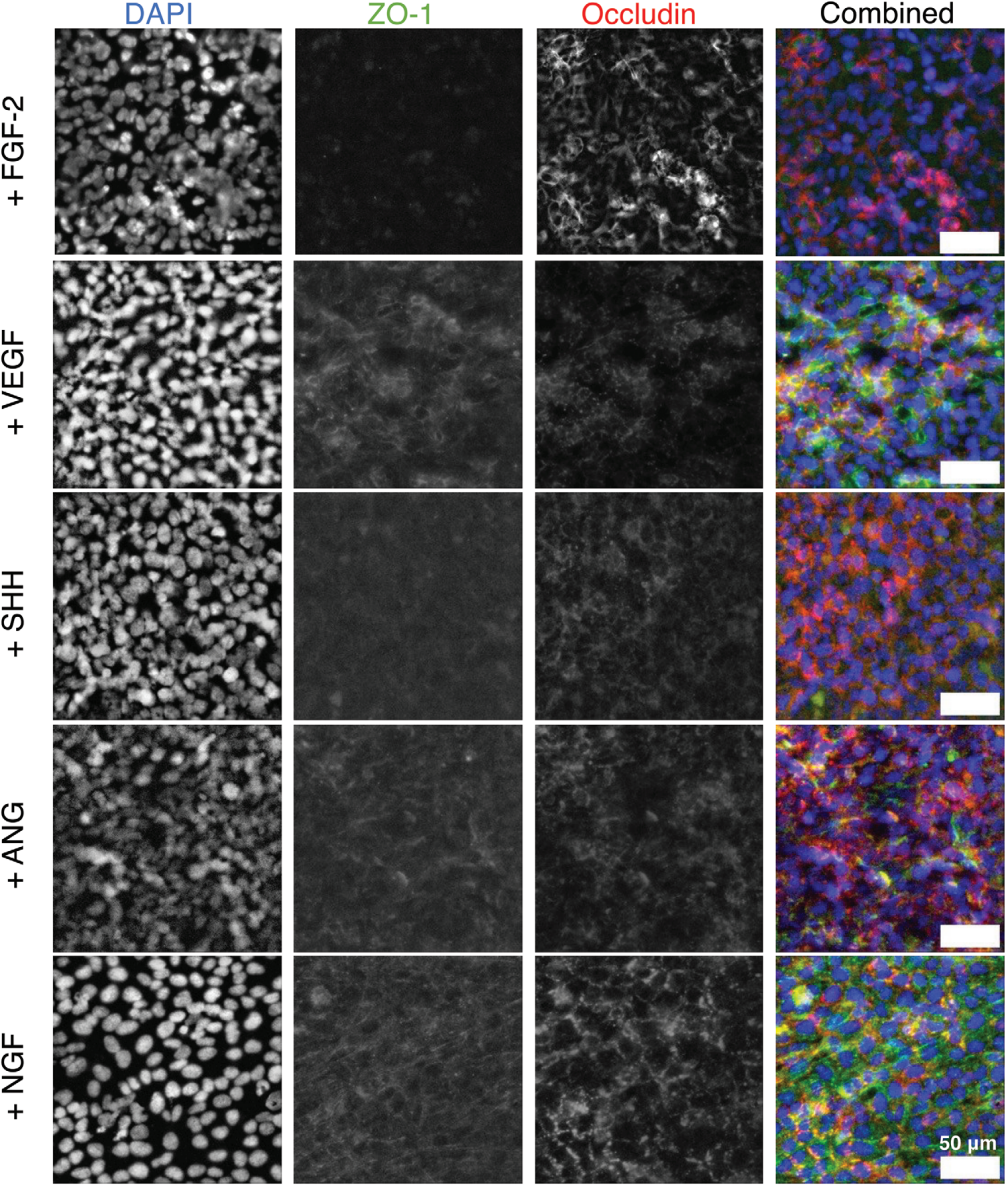
Comparison of different growth factors on the expression of ZO‐1 and occludin immunofluorescence of hCMEC/D3 cells cultured on PLLA‐pPEA‐FGF‐2 membranes. hCMEC/D3 cells were cultured for 10 days on membranes.

After confirming the ability of PLLA‐pPEA‐Fn electrospun membranes to drive changes in hCMEC/D3 cells, with respect to the BBB, a panel of potentially barriergenic growth factors was selected, and again shown in both commercial high FGF‐2 concentration medium and low FGF‐2 medium. Tight junction proteins are considered essential in the formation and function of the BBB, and therefore their expression and correct localization are used as benchmarks within BBB in vitro modeling, as it is shown to affect barrier characteristics, such as decreased permeability.^[^ ^52^, ^53^ ^]^ The occludin and ZO‐1 expression were visualized within their cells to establish whether the increased expression was cytoplasmic or localized to cell–cell junctions for the panel of growth factors tested (**Figure** **9**). The no ‐GF control shows low level of ZO‐1, mainly intracellular, although high occludin signal with areas of significant cell–cell junction localization. The FGF‐2 samples showed the highest level of ZO‐1 and occludin signal although this does not show clear cell junction localization. Interestingly, nuclei are also smaller and closer packed than in the no growth factor control. Of all the growth factors tested, NGF showed the most promising results and remarkably also displayed larger nuclei than the other conditions tested. The relationship between nucleus size and localization of ZO‐1 and occludin to cell–cell junctions would be of interest for future studies. Furthermore, the NGF condition showed a higher cell density. This reportedly results in a greater number of cell–cell contacts, and is shown to reduce metabolic activity within a cell monolayer, which itself is a characteristic of BMECs.^[^ ^54^ ^]^ In the future, the metabolic activities of monolayers seeded on different GF membranes would be an interesting avenue to investigate.

**Figure 10:**
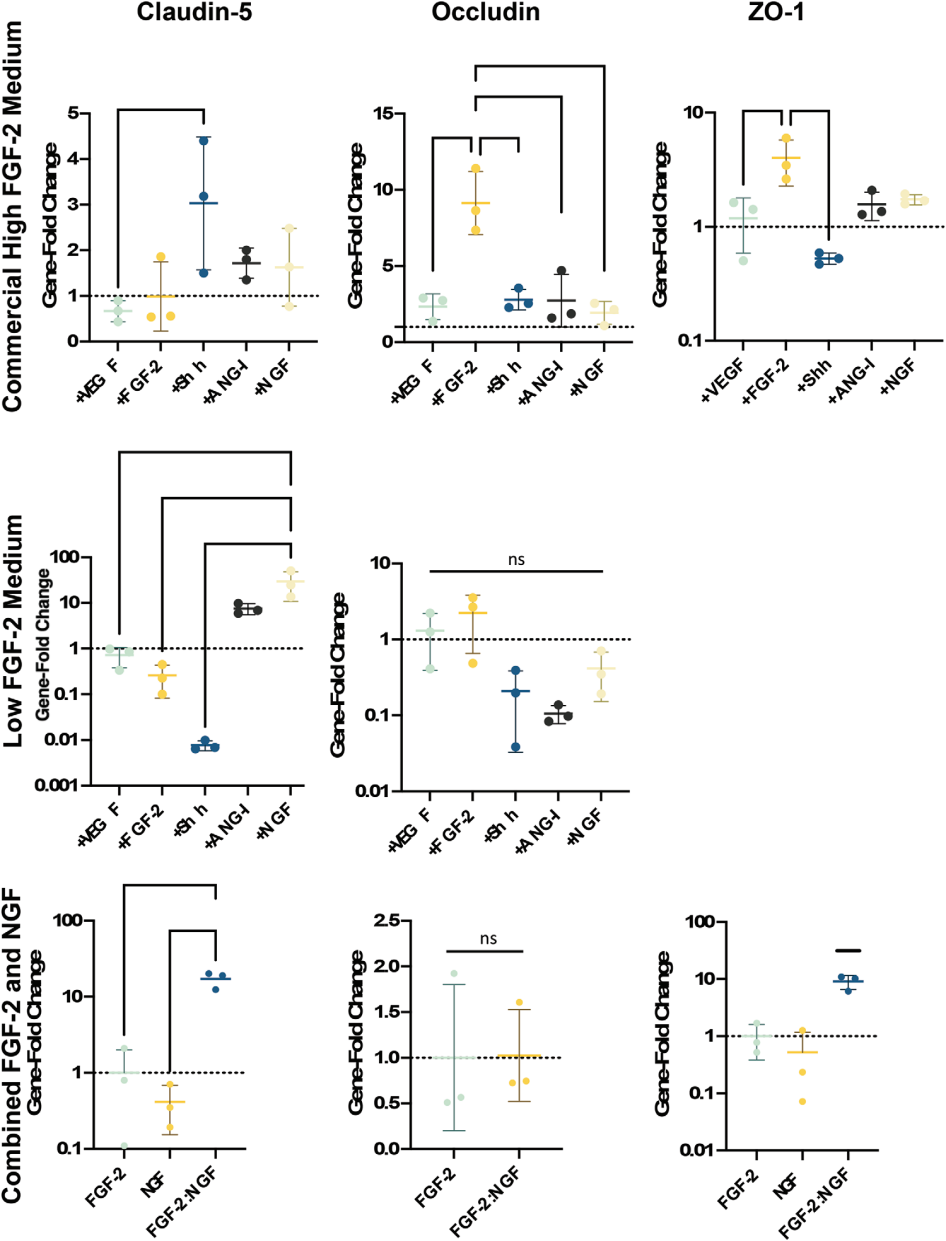
Comparison of gene expression of TJ and TJ‐associated proteins in response to different growth factors. Results on the top row are results from hCMEC/D3 cells cultured in high FGF‐2 concentration commercial cell medium, the middle row and bottom row are results acquired from hCMEC/D3 cells grown in low FGF‐2 concentration medium. Results from the bottom row additionally show cells grown on PLLA‐pPEA‐Fn membranes with FGF‐2 and NGF co‐adsorbed onto the surface. ^*^ *p* < 0.05, ^**^ *p* < 0.01, ^***^ *p* < 0.001.

**Figure 11:**
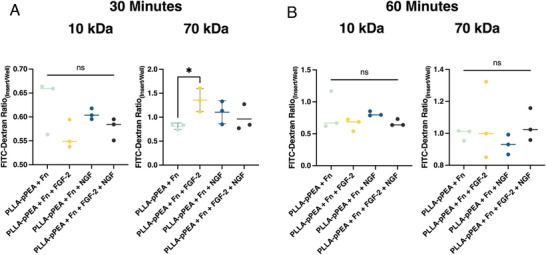
Effect of NGF, FGF‐2, and their combination of FGF‐2 and NGF on barrier permeability. Permeability is quantified as the ratio of FITC‐dextran signal between the insert and the well at 30 and 60 min after adding either 10 kDa or 70 kDa FITC‐dextran to the insert. Panels (A) and (B) illustrate the effect of NGF, FGF‐2, and their combination on barrier permeability at 30 and 60 min, respectively. ^*^ *p* < 0.05.

Beyond visualization, the gene expression of TJ proteins, claudin‐5 and occludin, were also evaluated (Figure 9). Within the high FGF‐2 cell media, sonic hedgehog (Shh) significantly increased the expression of claudin‐5 in comparison to other growth factors and the no‐growth factor control. In low FGF‐2 conditions, the Shh effect is sequestered, and instead only NGF significantly increases expression in comparison to the control condition and other growth factors, aside from Ang‐I, which is of interest as both Ang‐I and SHH are stated to improve barrier characteristics, although in the case of Ang‐I, this may lie in its ability to antagonize VEGF (**Figure** **10**).^[^ ^55^, ^56^, ^57^ ^]^ Shh is similarly linked to the recovery of the barrier function after disease and disruption, and may therefore not play a role in initial barrier formation.^[^ ^58^ ^]^ Occludin expression is highest in FGF‐2 condition for both low and high FGF‐2 cell media expression, although this is only statistically significant in the low FGF‐2 condition. A comparison was conducted between FGF‐2 and NGF and their combined effect in light of their positive results. In combination, NGF and FGF‐2 significantly increased the expression of both claudin‐5 and occludin, in reduced FGF‐3 concentration growth medium, although again neither growth factor significantly affected occludin expression (Figure 10). However, despite the increase in claudin‐5 and occludin mRNA expression, when the effect on barrier permeability was tested 10 days post cell seeding on PLLA‐pPEA‐Fn‐FGF‐2, ‐NGF, and the combination of both growth factors, these did not have a significant effect on the permeability of our BBB model to 10 kDa and 70 kDa FITC‐dextran (**Figure** **11**). The only exception was observed in the permeability to 70 kDa FITC‐dextran, where a significant decrease was noted when comparing PLLA‐pPEA‐Fn with the addition of FGF‐2 after 60 min (Figure 11). These results do not align with the mRNA and immunofluorescence findings, suggesting a potential delayed maturation of the in vitro barrier in our model. This delay might have prevented the increased expression of TJ‐protein mRNAs and the visible protein expression from forming mature intercellular links. Additionally, there may be significant effects on permeability not observed due to the limitation of testing only two molecular weights of dextran (10 and 70 kDa). We propose extending the cell incubation period to 14 or 20 days in future experiments, with multiple permeability testing time points in between, to better understand the barrier maturation process using fibronectin‐coated PLLA‐pPEA membranes. As of now, these results show a promising *amuse‐gueule* to future studies evaluating the minimum necessary combination of growth factors for the formation of the BBB in vitro.

**Figure 12:**
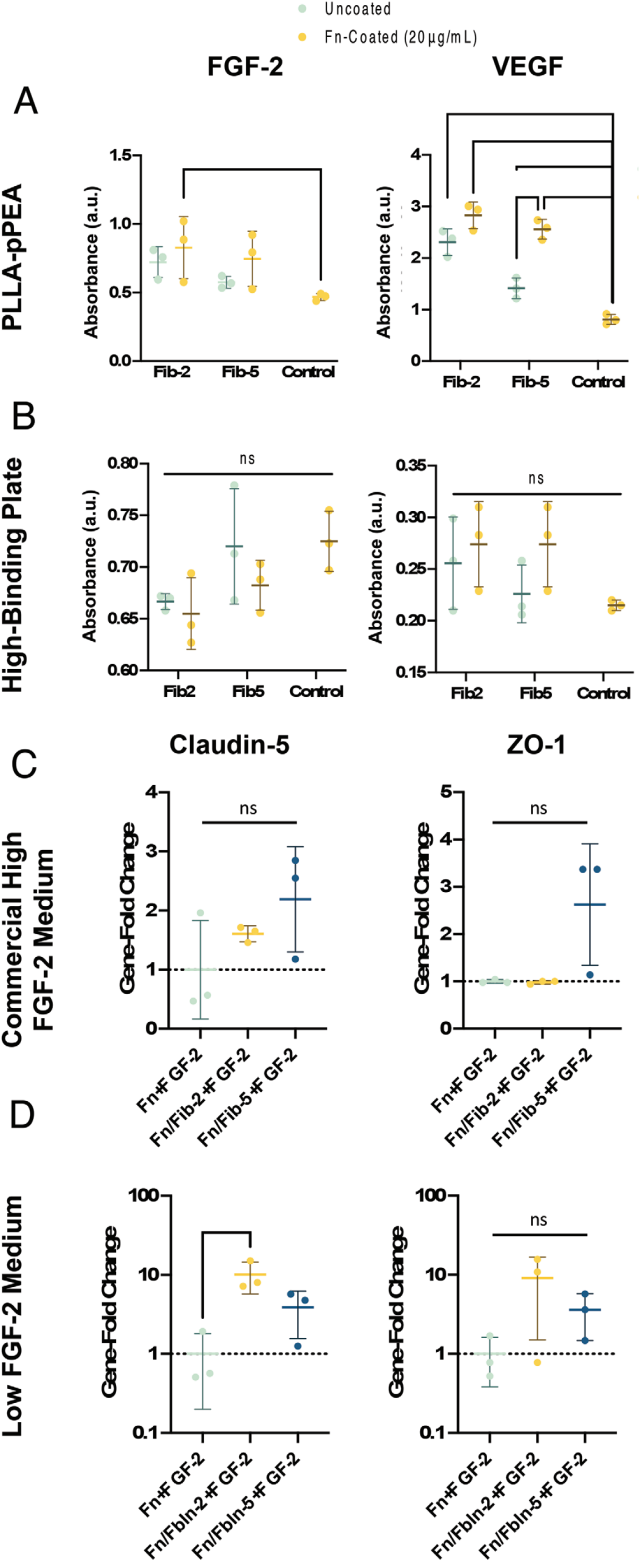
Incorporation of fibulin‐2 and fibulin‐5 into the in vitro model for the improvement of barrier characteristics. A) Sandwich ELISA to evaluate growth factor retention on PLLA‐pPEA membranes of fibulin‐2 and fibulin‐5 with and without fibronectin for FGF‐2 (left) and VEGF (right). B) The results of ELISAs against FGF‐2 (left) and VEGF (right) growth factor retention on fibulin‐2 and fibulin‐5 with and without fibronectin after immobilization of the ECM proteins on high‐binding plates. Claudin‐5 and ZO‐1 expression of hCMEC/D3 cells seeded on PLLA‐pPEA membranes for 10 days with either Fn+FGF‐2, Fn/fibulin‐2+FGF‐2, or Fn/fibulin‐5+FGF‐2 in C) high FGF‐2 concentration medium and D) low FGF‐2 concentration medium. ^*^ *p* < 0.05, ^**^ *p* < 0.01, ^***^ *p* < 0.001.

As a potential method to improve the model, fibulin‐2 and fibulin‐5, which bind Fn in vivo, were co‐adsorbed with Fn. While there is tentative evidence in the literature for the ability of fibulins to bind GFs, fibulin‐5, in particular, has been shown to have a strong inhibitory effect on angiogenesis (**Figure** **12**).^[^ ^59^, ^60^, ^61^, ^62^ ^]^ Due to this, its ability to perhaps enhance the barrier formation was considered. Fibulin‐2 and 5 showed an increase in VEGF and FGF‐2 binding specifically on pPEA‐coated PLLA membranes, rather than high‐affinity plates, particularly fibulin‐2. Microscale thermophoresis (MST) assays were performed with fibulin‐2 against a panel of GFs and Fn to establish whether they potentially bind each other. Of these, only VEGF showed potential as a fibulin‐2 binding partner. To evaluate whether fibulin‐2 and fibulin‐5 would affect the barrier formation of hCMEC/D3 cells in vitro, the gene expression and IF of TJ and TJ‐associated proteins were acquired. While no changes were seen in the IF data, Fn‐ Fibulin‐5 condition showed the greatest increase in ZO‐1 and claudin‐5 expression. When VEGF was used in combination with media containing high FGF‐2 content, we do not see increased expression of tight junctions which is compatible with the formation of barriers with lower tightness. Fibulin‐5 and −2 therefore have the beneficial effect of both increasing VEGF binding of pPEA‐Fn, but additionally improve the barriergenic potential of the model.

## 3 Conclusion

In summary, our study has successfully highlighted the beneficial impact of the pPEA‐Fn coating of electrospun membranes in the development of a minimalistic in vitro model of the blood–brain barrier. We have shown the potential and effectiveness of this novel PLLA‐pPEA‐Fn‐based electrospun membranes. By combining the positive mechanical properties and processibility of PLLA and the spontaneous material‐driven fibrillogenic capabilities of pPEA, we designed and characterized electrospun membranes with the aim of achieving a high similarity to the native collagenous BM with respect to both the nanofiber diameter, pore size, and membrane thickness. The membranes showed an enhanced growth factor retention and improved accessibility of the heparin binding site, and showed a decreased permeability compared to the same coating conditions to hCMEC/D3 cells grown on Transwell inserts. The fibrous nature of electrospun membranes plays a crucial role, as evidenced by the significant increase in BBB‐related protein gene expression observed on pPEA‐coated electrospun membranes in comparison to MilliCell cell culture inserts. Furthermore, our investigation into the effects of different growth factors and ECM proteins on barriergenesis in immortalized BMECs revealed the barrier‐forming results of NGF and FGF‐2 when presented bound to the membranes using the pPEA‐Fn system. Combining NGF and FGF‐2 had a synergistic effect on gene expression, surpassing our expectations. In spite of this, these growth factors did not significantly decrease permeability in the in vitro BBB model, potentially due to insufficient time given for barrier maturation. The only exception was a significant reduction in permeability to 70 kDa FITC‐dextran with pPEA‐PLLA‐Fn and FGF‐2 after 60 min. Finally, we demonstrated the improvement to VEGF retention when fibulin‐2 is co‐adsorbed with fibronectin, and overall improvement to barrier properties with the addition of fibulin‐5 to the PLLA‐pPEA‐Fn‐FGF‐2 in vitro BBB model. Overall, our novel in vitro model platform also provides a promising basis for the further exploration of key BBB barriergenic signals and offers exciting prospects for future research in the field of blood–brain barrier in vitro model development.

## 4 Experimental Section

### Preparation of Solutions and Electrospinning

PLLA (8% w/v) nurdles (Goodfellow Cambridge, UK), were dissolved in hexafluoro‐2‐propanol (HFIP) (Alfa Aesar, USA) at room temperature under stirring conditions. All electrospinning was performed using a custom‐built electrospinning set‐up. Membranes were electrospun onto a collector with aluminum foil at a constant rate of 0.9 mL min^−1^ using a 1 mL syringe and needle with 0.15 mm internal diameter at a gap distance of 20 cm unless otherwise stated, and applied voltage of 15 kV. For traction force microscopy, FluoSpheres microspheres (Thermo Fischer Scientific, USA) (580/605 nm) were diluted in the PLLA (8% w/v) HFIP solution in a 1:100 000 dilution, achieved by diluting stock in Dulbecco's phosphate‐buffered saline (DPBS) in series of dilutions under sterile conditions. All electrospun membranes were loaded onto custom‐designed 3D‐printed cell culture inserts.

### Plasma Polymerization

Plasma equipment setup was as described in previous publications.^[^ ^24^, ^31^ ^]^ Electrospun membranes plasma coated within a custom‐built plasma chamber, which was brought to the operating pressure of 0.015–0.020 mbar. Membranes were initially cleaned, with residual organic matter removed, through air plasma treatment for 5 min. This was followed by ethyl acrylate monomer (Sigma–Aldrich, USA) plasma for 15‐min. The operating radio frequency incident power for both plasma treatments was 50 W.

### Protein Adsorption

Fibronectin (Sigma–Aldrich, USA) was adsorbed onto surfaces at a concentration of 20 µg mL^−1^ in DPBS. Samples were then washed once with DPBS. If laminin (Biolaminina, Sweden) or fibulin (R&D Systems, USA) were used, they were co‐adsorbed with fibronectin at a final concentration of 5 nmol. All growth factors used were purchased from R&D Systems and adsorbed onto membranes at a final concentration of 100 ng mL^−1^ in DPBS, unless otherwise stated.

### Scanning Electron Microscopy and Analysis of Nanofibers

Scanning electron microscopy images were obtained with the iii Imaging Facility, University of Glasgow (JEOL6400 SEM, JEOL, Japan), which was performed at an accelerated voltage of 10 kV. ImageJ was used to determine fiber diameter and pore size by measuring the fiber diameters of the uppermost ten fibers of each image. Pores were defined as the gap between nanofibers in the same focal plane and measured as the greatest distance from fiber to fiber for each pore.

### Trans‐Endothelial Electrical Resistance (TEER)

TEER was performed using an EVOM epithelial Voltmeter with an EndOhm chopstick electrode (World Precision Instruments, INC, Sarasota, FL). Samples were left at room temperature for 5 min prior to measurements to ensure comparable readings between all conditions.

### Transmission Electron Microscopy and Analysis of Membrane Thickness

Transmission electron microscopy images were obtained with the iii Imaging Facility, University of Glasgow (JOEL1200 EX II Transmission Electron Microscope, JEOL, Japan) following the embedding of membranes in Epon resin. TEM images were analyzed using ImageJ software to measure membrane thickness, which was measured at five locations per image over a minimum of five cross‐sections.

### Surface Chemical Composition with X‐Ray photoelectron spectra

X‐ray photoelectron spectra were obtained through the National EPSRC Users’ Service (NEXUS) at Newcastle University, obtained using the K‐Alpha apparatus (Thermo Scientific) with a microfocused monochromatic Al Kα source (X‐ray energy = 1486.6 eV) at a voltage of 12 kV, current of 3 mA, power of 36 W, and spot size of 400 µm × 800 µm. Spectra analysis and curve fitting were performed using CasaXPS software version 9.

### Atomic Force Microscopy

Atomic force microscopy was used to quantify membrane stiffness. AFM nanoindentation (AFM/FS, Nanowizard‐3, JPK, Germany) was performed in solution on membranes immersed in DPBS, which had previously been immersed for a minimum of 1 h in DPBS, with cantilevers (30‐40 N m^−1^, Bruker, USA) were functionalized with microbeads (20 mg mL^−1^, size 20 µm, monodisperse, Corpuscular Inc., USA) in force spectroscopy mode. Membranes were indented at a minimum of 500 nm and assessed using constant force over 2500 µm^2^, for a total of 25 measurements with at least five maps per replicate. The analysis was performed using JPKSPM processing software using the Hertz model for a spherical indenter to fit the curves obtained.

### Traction Force Microscopy

To quantify the force magnitude required of cells to rearrange uncoated and pPEA‐coated PLLA electrospun membranes, Flourosphere electrospun fibers were seeded with mesenchymal Stem Cells (MSCs) at a seeding density of 100 cells/membrane 24 h prior to imaging. LIVE/DEAD kit Calcein‐AM was added to cell media 20 min prior to imaging so that the final dilution was 1:5000 to better visualize MSCs on electrospun membranes. Samples were imaged with an EVOS FL Auto Imaging System (ThermoFisher Scientific, USA) with incubator function set to 37 °C for the duration of the image acquisition with cells. A minimum of ten cells were imaged per membrane, with four membranes per condition. After image acquisition cells with Flourospheres, media was replaced with 0.5 wt% sodium dodecyl sulfate (SDS) for 5 min to remove cells, after which cell locations were imaged again to obtain Flourosphere displacement. The traction forces were calculated using ImageJ using the protocol and plugins developed by Tseng.^[^ ^63^ ^]^ The final image was edited in ParaView to edit the images to use a universal scale.

### ELISA for Protein Adsorption Quantification

Indirect ELISAs were performed to quantify fibronectin adsorbed onto membranes. Following 1 h of fibronectin adsorption, membranes were washed with DPBS and blocked with 1% bovine serum albumin (BSA) in DPBS. After washing, primary antibody P5F3 (Santa Cruz Biotechnology, USA) was added in DPBS at a final dilution of 1:2000 for 1 h under agitation, followed by washes. Secondary antibody goat‐anti‐mouse‐HRP (Invitrogen, USA) was added for 1 h under agitation. Substrate solution (1:1 V:V Reagent A: Reagent B) (R&D Systems, USA) was added to the samples for 20 min and then stopped by adding stop solution (4 N sulfuric acid) (R&D Systems, USA). The supernatant from the membranes was then removed and added to the ELISA plate as technical triplicates and read at 450 and 540 nm using a plate reader (BIOTEK, USA). Sandwich ELISAs to quantify FGF‐2 and VEGF adsorption onto membranes were performed using the ELISA DuoSet FGF‐2 and VEGF (R&D Systems, USA) kits, respectively, according to the manufacturer's protocol. FGF‐2 and VEGF were adsorbed onto membranes for 1 h, after which the supernatant was removed and processed using the ELISA DuoSet protocols. Adsorption was quantified by comparing absorbance between supernatant removed from high‐affinity plates or membranes and pre‐adsorption solutions.

### Cell Culture

All cells were incubated at 37 °C in 5% CO2 and split when 70% confluent.

hMCEC/D3 cells (Merck) were used between passages 1 and 15 for blood–brain barrier in vitro modeling. Cells were cultured on vessels coated in a 1 in 20 ratio of rat‐tail collagen type I in DPBS for a minimum of 1 h at 37 °C prior to cell seeding, with collagen solution being carefully aspirated and vessel washed with DPBS prior to cell addition. Cells were seeded on electrospun scaffolds at 200 000 cells cm^−1^ for monolayer‐achieving experiments, and 4000 cells cm^−2^ for individual cell experiments lasting 24 h, unless otherwise stated. Commercial cell growth medium/high FGF‐2 medium consisted of the Endothelial Cell Growth Medium MV with supplement mix (PromoCell, Germany). Low FGF‐2 cell growth medium comprised of MCDB 121 medium (Gibco, USA) supplemented with a final concentration of 5% fetal bovine serum, 1X GlutaMax, 1X lipid supplement 10 µg mL^−1^ ascorbic acid, 550 nM hydrocortisone, 2.5 µg mL^−1^ insulin‐transferrin‐selenium, 100 µg mL^−1^ heparin, 50 µg mL^−1^ gentamycin, and 1 ng mL^−1^ FGF‐2.

Mesenchymal Stem Cells, MSCs, (Promocell) were used for TFM experiments and used at passage 5. Seeding density was 100 cells per membrane and grown in Dulbecco's modified eagle's medium (DMEM) (Gibco, USA) supplemented with a final concentration of 10% fetal bovine serum, 2 nM glutamine, 1% minimum essential medium non‐essential amino acids, and 1% in house antibiotic mix of penicillin‐streptomycin.

### Immunofluorescence

hCMEC/D3 cells were fixed with ice‐cold ethanol and permeabilized with 1% Tween 20 in DPBS. Imaging for ZO‐1 and occludin was performed using the primary antibody anti‐ZO‐1 (Thermo Fischer Scientific, USA) and anti‐occludin (Proteintech, USA). Secondary antibodies used were Cy3‐anti‐rabbit (Jackson Immunoresearch, USA) and 488‐goat‐anti‐mouse (Thermo Fischer Scientific, USA). Images were taken at 40X in the ZEISS AxioObserver Z.1.

### qRT‐PCR Gene Expression and Analysis

Total RNA was extracted using a Qiagen RNeasy micro kit according to the manufacturer's protocol. The resulting RNA concentration was measured using nanodrop and normalized for cDNA preparation using the Qiagen Quantitect reverse transcription kit (Applied Biosystems, USA) according to the protocols published by the manufacturers. The Qiagen Quantifast SYBR green qRT‐PCR was used to perform amplification with the primers (Eurofins Genomics, Germany) listed in **Table** **1** below using the Applied Biosystems 7500fast Real Time PCR system. All gene expression levels were standardized using GAPDH internal control and quantified using the ΔΔCt method, represented as a gene‐fold change to experimental controls.

**Table 1:** qRT‐PCR primer targets and sequences used.

Target	Forward Sequence (5′‐3′)	Reverse Sequence (5′ −3′)
GADPH	ACGGATTTGGTCGTATTGGG	ATTTTGGAGGGATCTGCTC
Claudin‐5	TCCTTAGTCCATGGCTAG	TTTTGGAGAGAGTTCAAACC
Occludin	GGACTGGATCAGGGAATATC	ATTCTTTATCCAAACGGGAG
ZO‐1	GAGATGGCAATATTCAAGAAGG	AGGGACATTCAATAGCGTAG
JAM‐A	GAAGGAGAATTCAAACAGACC	AAACACATCCGAAGAAGTAG

### In Vitro Barrier Permeability Analysis

To evaluate barrier permeability, a permeability assay on day 10 post cell seeding was performed with two weights of FITC‐dextran (Invitrogen, USA) separately: 10 and 70 kDa. Both solutions were prepared to a final concentration of 1 mg mL^−1^ in low FGF‐2 cell media. The cell media from cell‐seeded electrospun membranes and Transwell inserts was aspirated, and the inserts were placed in fresh wells containing 800 µL of media. Then, 200 µL of FITC‐dextran cell media was added to the inserts. After a 30‐min incubation, 100 µL of solution was taken from both the apical and basolateral compartments and returned to the incubator for an additional 30 min before the final measurement was taken at the 60‐min mark. Samples were protected from the light before readings on fluorescence measures were taken. (**Table** **2**).

**Table 2:** Comparison of the literature coverage on the relationship between FGF‐2 and the BBB, and NGF and the BBB.

GF	Finding	Reference
FGF‐2	Glioblastoma cells secrete FGF‐2 when in contact with BBB in vitro, strengthening barrier properties.	[64]
	FGF‐2/−5 double knock out mice have increased BBB permeability.	[65]
	In vitro, 0.5 – 5 ng mL^−1^ FGF‐2 improves barrier properties.	[66]
	Endogenous FGF‐2 found in cytoplasm of invading capillaries but not adult counterparts.	[67]
	FGF‐2 has a protective role in the BBB.	[68]
	FGF‐2 moderately increases expression of GLUT‐1.	[69]
NGF	Brain endothelial cells proliferate in response to NGF.	[70]
	NGF promotes VEGF expression in corneal endothelial cells.	[71]
	NGF promotes FGF‐2 expression in human chondrocytes.	[72]

### Statistical Analysis

Statistical analysis was performed using the GraphPad Prism 9.02 software. A statistically significant number of repetitions were performed for each measurement, usually three unless otherwise stated.

Standard deviations were presented as error bars in graphs. The goodness of fit of all datasets was assessed via D'Agostino‐Pearson Normality test. Statistical analysis was performed using either analysis of variant (one‐way and two‐way ANOVA) and Student's *t*‐test. Statistical significance was considered when *p* < 0.05.

## Conflict of Interest

The authors declare no conflict of interest.

## Data Availability

The data that support the findings of this study are available from the corresponding author upon reasonable request.
